# Health and Sedentary Behaviors within Polish Nurses: A Cross-Sectional Study

**DOI:** 10.3390/nu15061312

**Published:** 2023-03-07

**Authors:** Anna Bartosiewicz, Edyta Łuszczki

**Affiliations:** Institute of Health Sciences, Medical College of Rzeszow University, 35-959 Rzeszow, Poland

**Keywords:** nurses, health behavior, sedentary behavior, health promotion, public health

## Abstract

Health behaviors play a pivotal role in improving and strengthening health. Nurses, who constitute the vast majority of employees in the health sector, play a crucial role not only in treating disease but also in promoting and maintaining optimal health for themselves and society. The purpose of the study was to assess the level of health and sedentary behavior and the factors influencing them among nurses. A survey, cross-sectional study was conducted among 587 nurses. Standardized questionnaires evaluating health and sedentary behavior were used. The study utilized both single-factor and multifactor analyses, employing the linear regression method and Spearman correlation coefficient. The results showed that the health behaviors of the survey nurses were at an average level. Sedentary time (in hours) was an average of 5.62 h (SD = 1.77) and correlates significantly (*p* < 0.05) and negatively (r < 0) with health behaviors in terms of the positive mental attitude subscale; the longer the sitting time, the lower the intensity of this type of health behaviors. The efficient functioning of the healthcare system is greatly dependent on nursing staff. To improve health behaviors among nurses, systemic solutions such as workplace wellness programs, incentives for healthy behaviors, and education on the benefits of a healthy lifestyle are needed.

## 1. Introduction

In recent years, there has been growing public awareness of the importance of health behaviors in sustaining and improving overall health [1,2,3,4,5]. Health behaviors include a variety of habits, attitudes, and intentional behaviors that can be pro- and anti-health and relate to the bio, psycho, and social aspects of human functioning [6,7,8]. Studies indicate the important role of proper nutrition, physical activity, participation in preventive examinations, maintaining safety, adequate sleep, and avoiding addictions (tobacco, alcohol, medications) [8,9,10]. Furthermore, an increasing amount of research indicates that a sedentary lifestyle not only significantly reduces quality of life but also leads to the development of many diseases, such as overweight and obesity, diabetes, hypertension, and spinal degeneration. Ultimately, it can even lead to significant disability and even death [11,12,13]. Sociodemographic factors, family circumstances, or type of work can influence health behavior [8,9]. Nurses, who constitute the vast majority of employees in the health sector, participate not only in combating the effects of the disease but also in every action leading to maintaining an optimal state of their own and society’s health [14,15,16]. Moreover, as healthcare professionals, nurses can serve as positive role models for their patients, families, and friends [16,17].

Unfortunately, numerous studies have indicated that employees in the healthcare sector, including nurses, often fail to practice behaviors that promote and sustain good health, despite their knowledge and high awareness of health [18]. It is difficult to pinpoint a clear cause. The issue is multifaceted and influenced by various factors. The unique nature of nursing work must be considered, including the shift system and the disruption of natural sleep, rest, and nutrition cycles, as well as overwork, stress, and time pressure [19,20]. These factors can make it challenging for nurses to maintain healthy habits [21]. Therefore, it is important for nursing leaders, in collaboration with healthcare organizations, to prioritize promoting and strengthening health behaviors among nurses [22].

This is not only a workplace health problem but also a potential financial and patient safety issue. Creating policies and jobs that take into account holistic support for this professional group is a guarantee of stress reduction, professional burnout, better health condition, and thus patient safety and high-quality healthcare [18,23,24].

The efficient functioning of the healthcare system is greatly dependent on nursing staff [14]. Staff shortages and the age of over 50 of the majority of nurses currently working in the system require special attention when discussing the importance of good health conditions of this professional group for public health. In addition, the fact that among the leading causes of death, most are related to behavioral factors increases the need for research in this field [25,26].

The purpose of the study was to assess the level of health and sedentary behavior and the factors influencing them among nurses.

## 2. Materials and Methods

This is a cross-sectional descriptive study, carried out from April to June 2021 in the Subcarpathian region in Poland. The study involved 587 nurses working in the following medical entities: primary health care, hospitals, outpatient specialist care, hospices, independent health care institutions, health care centers and social welfare homes, private sector, nursing homes, and resorts. Medical entities have been randomly selected through a randomized algorithm program. The sample size was determined using EPI INFO (StatCalc) software. A multistage random cluster sampling method was used.

Convenient sampling was used, and the inclusion criterion for the participants was professionally active nurses with a license to practice, at least 2 years of work experience, and consent to participate in the study. The questionnaire was distributed to participants in sealed envelopes to ensure confidentiality. In total, 1150 questionnaires were distributed, 614 were collected. All questionnaires were checked, and due to the incompleteness of the answers, 27 questionnaires were excluded from the study. Data from 587 questionnaires were entered into an Excel spreadsheet, coded, and subjected to statistical analysis. To explain the purpose and conditions of the study, a meeting was organized in the medical entities before the survey. Participation in the study was voluntary, with the possibility of withdrawing from the study at any stage and without consequences. The nurses were assured of the anonymity of the study. The nurses included in the study were a representative group of all nurses working in the southeastern region of Poland (the error threshold was 4%, that is, the test power was 0.96).

The survey technique used a standardized questionnaire, including the Health Behaviors Inventory (HBI), a modified Polish version of the International Physical Activity Questionnaire (IPAQ), and questions regarding the sociodemographic data of the respondents.

### 2.1. Health Behaviour Inventory

The HBI questionnaire is used to monitor pro-health behaviors; it contains 24 statements describing health-related behaviors. Analysis of the frequency of individual behaviors indicates the overall result of individual health behaviors.

The questionnaire is divided into four subscales:

- Proper Eating Habits (PEH), which primarily take into account the type of food eaten (type of bread, fruit, and vegetables);
- Preventive Behaviours (PB), related to compliance with health recommendations and how to obtain information in the field of health and disease;
- Health Practices (HP), related to daily habits related to sleep, physical activity, and recreation;
- Positive Mental Attitude (PMA), related to psychological factors such as avoiding stress, strong emotions, or other depressing situations.

Respondents specifying the frequency of specific health behaviors answer questions on a five-point scale, where 1 means almost never, 2 means rarely, 3 means occasionally, 4 means often, and 5 means almost always. The questionnaire evaluates the frequency of behaviors over the last year, and the average examination time does not exceed 5 min.

To obtain a general indicator of the intensity of health behaviors, all values marked by the respondent are summarized. The number of points varies from 24–120. Higher scores correspond to a higher level of declared health behaviors. The results are interpreted according to the sten scale: according to the norms (separate for women and men) given in the key to this questionnaire, sten scores 1–4 mean low, sten scores 5–6 average, and sten scores 7–10 high regarding increased health behaviors. The results of each of the four subscales of the HBI questionnaire are the averages of the responses given to the questions included in them. The results obtained were interpreted as low, high, or medium according to the following criteria: an average of 1 means “almost never”; an average of 2 means “rarely”; an average of 3 means “from time to time”; an average of 4 means “often”; and an average of 5 means “almost always” [27].

### 2.2. Sedentary Behaviors (SB)—Single Item of the IPAQ

Respondents retrospectively reported their sedentary behaviours (SB). To obtain the most accurate results, before the survey, nurses were asked to pay attention to and/or record how much time they spend sitting during the day before respondents retrospectively reported their sedentary behavior in the survey. A single item questionnaire (the short version of the IPAQ questionnaire) was used. The question asked: “How long have you been sitting in the last 7 days? When answering, nurses indicated how much time (in hours per day) they spent sitting [28].

### 2.3. Statistical Methods Used

The analysis of quantitative, which represent numerical, data was performed by calculating the mean, standard deviation, median, and quartiles. The analysis of qualitative variables (i.e., not expressed in numbers) was performed by tabulating the frequency and percentage of each value. Single- and multi-factor analysis of the influence of many variables on the quantitative variable was performed using the linear regression method. The results are presented as values of the parameters of the regression model with a 95% confidence interval.

The correlations between the quantitative variables were analysed using the Spearman correlation coefficient. The analysis adopted a significance level of 0.05. Therefore, all *p*-values less than 0.05 were interpreted as correlations that are unlikely to have occurred by chance.

The analysis was performed in the R program, version 4.1.3 [29].

## 3. Results

### Characteristics of Study Group

The study involved 587 nurses, mostly women (80.75% vs. 19.25%), aged 38–52. Details are presented in Table 1.

## 4. Findings

The results showed that almost half (41.74%) of the participants declared an average level of health behaviors, 33.39% were high and 24.87% were low (Table 2 and Table 3).

The average results of the subscales “Proper eating habits”, “Preventive behaviors” and “Positive mental attitude” were within the range of 4 in a scale of 1–5, where 4 indicates a high frequency of behaviors in these areas. The average result of the “Health practices” subscale was 3.39 points (rounded to 3). Thus, the average frequency of undertaking behaviors in this area is “from time to time” (Table 4).

Univariate and multivariate analyses in the linear regression model for the entire HBI scale (separate for each of the analyzed features) showed that none of the variables analyzed in the study are a significant predictor of the score on this scale (*p* > 0.05), (Table 5).

The single-factor linear regression model in the scope of the PEH subscale (separate for each of the features analyzed) showed that an important (*p* < 0.05) predictor of health behavior among nurses was the level of education, specifically bachelor’s title (regression parameter is 0.249, so increases the result on this scale by an average of 0.249 points in relation to secondary education). The multivariate linear regression model within the scope of the PEH subscale showed that important (*p* < 0.05) independent predictors of the result of health behavior among nurses were the level of education, specifically the bachelor’s title (the regression parameter is 0.314, and therefore increases the result on this scale, on average, by 0.314 points in relation to secondary education), and the workplace—that is, work in IHI (the regression parameter is 0.239, and thus increases the result on this scale by an average of 0.239 points in relation to the lack of employment in IHI), (details available in Supplementary Materials—Table S1).

The single and multivariate linear regression model (separate for each of the analyzed features) in the scope of the PB subscale showed that none of the variables analyzed in the study features is an important predictor of the result on this scale (*p* > 0.05), (details available in the Supplementary Materials—Table S2).

The single-factor linear regression model (separate for each of the analyzed features) in the scope of the PMA subscale showed that none of the analyzed features are an important predictor of the result on this scale (*p* > 0.05). The multivariate linear regression model in the scope of the PMA subscale showed that important (*p* < 0.05) independent predictors on this scale are age (the regression parameter is 0.026, so each subsequent year raises the result on this scale by 0.026 points) and nurses’ work experience (the regression parameter is −0.025, so each subsequent year of work reduces the result on this scale by an average of 0.025 points), (details available in the Supplementary Materials—Table S3).

The single and multivariate linear regression model (separate for each of the analyzed features) in the HP scope showed that none of the variables analyzed is an important predictor of the result on this scale (*p* > 0.05), details available in Supplementary Materials—Table S4).

The results showed that sedentary time (in terms of hours) had an average of 5.62 h (SD = 1.77) and ranged from 1.71 to 11.29 h/day (Table 6).

The single-factor linear regression model (separate for each of the features analyzed) showed that the gender of the male is a significant (*p* < 0.05) predictor of sitting time among the nurses surveyed (the regression parameter is −0.573, indicating that it reduces sitting time, on average, by 0.573 h per day in relation to the female sex. Additionally, the number of full-time jobs is also a significant predictor (*p* < 0.05), with a regression parameter of −0.38. This indicates that each additional job reduces sitting time by an average of 0.38 h per day. The multivariate linear regression model showed that the gender of men is still a significant (*p* ˂ 0.05) independent predictor of sitting time, with a regression parameter of −0.489. This means that it reduces sitting time by an average of 0.489 h per day with respect to the female sex (Table 7).

Correlation results showed that sitting time correlates significantly (*p* < 0.05) and negatively (r < 0) with health behaviors, specifically with the positive mental attitude subscale. This means that the longer the sitting time, the lower the intensity of this type of health behavior (Table 8).

## 5. Discussion

It was noted that the health behaviours of nurses were at an average level. This is consistent with findings by Ross et al., who found that despite health knowledge and awareness, nurses do not always maintain healthy behaviors [18]. Nurses’ working conditions have been shown to affect their health, with numerous authors identifying this professional group as being burdened by health problems, such as obesity, insufficient or lack of physical activity, inappropriate eating behaviours, smoking, excessive alcohol consumption, and inadequate rest [21,30,31,32].

According to the 2022 Report of the Supreme Chamber of Nurses and Midwives, the numerically largest age range among nurses is 51–60 years, which includes 84,444 nurses, which represents 36.0% of the number of nurses employed in Poland. Despite having acquired pension rights, up to 68,955 nurses still work in the profession in the 61–70 age bracket and the over 70 age bracket. These groups represent 29.36% of the total workforce. By 2030, up to 65% of nurses currently employed are projected to be of pensionable age [33]. In our study, the mean age of the nurses in our study was 44.63 ± 9.24 years and their work experience was 19.95 ± 9.53 years.

The issues of health behaviors among nurses have been widely addressed in both Polish and foreign research [21,34]. In our study, we sought to analyze the amount of time nurses spend on sedentary behavior and how these behaviors affect their overall health, which we evaluated using the HBI questionnaire. The results showed that almost half (41.74%) of the participants declared a moderate level of health behaviors, while 33.39% reported a high level, and 24.87% reported a low level. This indicates that only about one third of the respondents have high health behaviors that may be related to their health status and the quality of their profession. On the contrary, the majority have moderate or low health behaviors. This is consistent with a study by Orszulak et al. who surveyed a group of nurses and also found that only 19.87% of the study group exhibited a high prevalence of health behaviors [34]. Moreover, the results of our studies are consistent with those of other authors. Książek et al. [35], the research by Różewicz et al. [36], and the study of Jankowska-Polańska et al. [37] all found that most examined nurses had a moderate level of health behaviors. Nurses face potential barriers to maintaining a healthy lifestyle both on and off the job. These include lack of breaks, shift work, and a fast pace of work. There is evidence that the incidence of excessive body mass among nurses is increasing [38]. In our study, the mean BMI was 25.28 ± 9.24, indicating overweight.

The multivariate linear regression model within the scope of the “positive mental attitude” subscale of HBI showed that the significant independent predictors on this scale are age and nursing job experience. Specifically, with age, the “positive mental attitude” has increased, and the work experience has decreased. Similar results were obtained in a study by Waksmaska et al., where statistically significant differences were shown in participants under 40 and over 50 years of age with respect to the level of health behaviors [39]. In the study by Orszulak et al., it was observed that there was an equally significant correlation between age and total HBI score, revealing a higher prevalence of each type of health behavior among older nursing staff [34]. The average work experience in our study was 19.95 ± 9.53 years. In an article by Jankowska-Polanska et al., a strong relationship was found between years of age and total HBI score. The group of respondents with more than 25 years of experience in the nursing profession had a higher health-behavior index than the nursing staff who worked for only 5 years [37]. However, in our study, we showed an inverse relationship, as the HBI subscale decreased with work experience. This may be related to job burnout, which researchers have frequently noted. However, the study by Górniak reported a significant decrease in positive psychological attitudes in respondents with more years of seniority [40].

The single factor and multivariate linear regression models within the scope of the “Proper eating habits” subscale showed that the significant predictor of health behavior among nurses was the level of education—specifically, having a bachelor’s degree. Our results showed that the higher the education level, the higher the level of health-promoting behaviors related to “Proper eating habits”. This finding is consistent with a study by Górniak et al., which found a statistically significant relationship between the level of education and the overall health behavior index [40]. Nursing staff with master’s and bachelor’s degrees reported a higher level of health-promoting behaviors than those with a secondary school or medical school degree. Similarly, Różewicz et al. [36] found that proper eating habits scored the highest in the group of nurses with a bachelor’s degree. Sedentary behaviors are defined as any wakeful behaviors that spend less than 1.5 metabolic equivalents while in a reclining, sitting, or lying position [41]. Several studies have measured nurses’ sedentary behavior and shown low levels of physical activity and high levels of sedentary behavior, with nurses spending up to 60% of their day in a sedentary state. Most of the nurses examined did not meet the physical activity guidelines. Nurses who work rotating shifts, 12-h shifts, and/or work full-time or part-time are at increased risk of physical inactivity [42].

Our study found that nurses spent an average of 5.62 h (SD = 1.77) per day being sedentary, with a range from 1.71 to 11.29 h. These results are consistent with those of Benzo et al. who reported that nurses spent 4.56 h of each 12-h shift sitting and 5.64 h of each 12-h shift standing [43]. The correlation analysis showed a significant negative association between sedentary time and positive mental attitude, indicating that higher levels of sedentary time were associated with lower intensity of positive mental attitude health behaviors. These findings are not surprising given that prolonged sitting can have negative effects on health [44,45]. While some studies have suggested that nurses lack motivation to adopt healthier lifestyles [46], others have highlighted environmental and occupational barriers [47]. Nevertheless, promoting lifestyle changes is crucial, particularly considering the aging nursing workforce, as well as the health issues associated with shift work, long working hours, and stressful work environments.

### Limitations

There are several potential limitations of this study that should be considered when interpreting the results. Firstly, the study had a limited geographic scope and there may be differences between departments of healthcare institutions. Secondly, the study was carried out during the COVID-19 pandemic, making it difficult to reach a larger group of respondents. It was also a very difficult time for nurses because they worked under difficult conditions. Thirdly, despite the assurance of anonymity, it was a self-assessment of specific health and sedentary behaviors, which may be partly subjective. Fourthly, the cross-sectional design of the study prevents us from establishing causality and temporality problems. Finally, sedentary behaviors were assessed based on the retrospective declaration of the nurses. Studies using more advanced and objective measuring devices, e.g., accelerometers, are needed. To increase the generality of the study, it should be extended and include more medical facilities in other regions.

## 6. Conclusions and Recommendations

The nurses in our study presented a moderate level of health behaviors, with the highest results in terms of preventive behaviors on the HBI scale and the lowest scores in terms of health practices. Age and work experience were found to affect nurses’ positive mental attitude, while level of education influenced proper eating habits. Additionally, the study found that the average sedentary behavior was 5.62 per day. To improve health behaviors among nurses, systemic solutions such as workplace wellness programs, incentives for healthy behaviors, and education on the benefits of a healthy lifestyle are needed.

Nursing leaders and employers play a pivotal role and can become advocates for system changes and be aware of the impact of social support and the hospital or unit culture on the ability of a nurse to practice health-promoting behaviors. They should create open work environments where nurses feel comfortable talking about workplace stressors and barriers to healthy behaviors, and thus support and promote nurses’ efforts to eat a healthy diet or exercise. When making staffing decisions, they should ensure that there is adequate support to provide meal and relaxation breaks for nurses. It should be noted whether canteens, coffee shops, and vending machines in medical facilities offer healthy food.

Because nurses themselves are best able to identify the needed programs and barriers in the workplace to healthy living, it is a good initiative to assess needs or hold focus groups to determine how best to encourage and support a healthy lifestyle. Holistic activities that promote health permanently, not only periodically, focusing on physical activity, healthy eating, and stress reduction, must be included in the nursing workplace. Both mindfulness-based stress reduction interventions and weekly yoga classes can be helpful in improving self-care and reducing nurse burnout. They are essential and should be activities that staff members can perform during the workday or immediately before or after their shifts.

A very positive example of initiatives taken in this area is the Healthy Nurses’ Nation Health campaign created by the American Nurses Association, which created an information website especially for nurses and thus supports and promotes pro-health behaviors among nurses [48].

Incorporating healthy behaviors into the nursing workplace is an investment that can not only provide immediate dividends for the nurses and staff, but long-term benefits for the organization and patients as well. In an age when it is imperative that all possible avenues for improving quality of care and decreasing healthcare costs be explored, interventions aimed at promoting the health, well-being, and performance of Polish nurses should be implemented to support nurses in making lifestyle changes and improving their health. Given the current shortage of nurses in Poland, this is of paramount importance. We hope that the findings of this study prompt further investigation in this area.

## Figures and Tables

**Table 1:** Characteristics of the study group.

Variable		Total (n = 587)
Age [years]	Average ± SD	44.63 ± 9.24
	Median	46
	Quartiles	38–52
Work experience [years]	Average ± SD	19.95 ± 9.53
	Median	21
	Quartiles	14–28
More than one full-time job	Average ± SD	1.45 ± 0.47
	Median	1,5
	Quartiles	1–1.75
Body Mass Index [kg/m^2^]	Average ± SD	25.28 ± 9.24
	Median	24.7
	Quartiles	21.9–27.2
Sex	Female	474 (80.75%)
	Male	113 (19.25%)
Education level	Basic nursing education	86 (14.65%)
	Bachelor	92 (15.67%)
	Master’s degree	409 (69.68%)
Place of work *	PHC	337 (57.41%)
	Hospital	210 (35.78%)
	IHI	43 (7.33%)
	OSC	54 (9.20%)
	Hospice	51 (8.69%)
	HCC/SWH	80 (13.63%)
	Long-term care	104 (17.72%)
	Private sector	96 (16.35%)
	Nursing home/resort	39 (6.64%)
Shift work and night duty	No	73 (12.44%)
	yes	514 (87.56%)
Self-assessment of health	Very good	84 (14.31%)
	Good	370 (63.03%)
	No opinion	100 (17.04%)
	Bad	33 (5.62%)
Participation in preventive examinations other than obligatory	No	446 (75.98%)
	Yes	141 (24.02%)
Chronic diseases	No	463 (78.88%)
	Yes	124 (21.12%)

* The value does not add up to 100, as multiple choice was possible; PHC—Primary Health Care; IHI—Independent Healthcare Institution; OSC—Outpatients Specialist Care; HCC—Health Care Center; SWH—Social Welfare Home; Long-term care.

**Table 2:** Level of Health Behavior Inventory among nurses (n = 587).

Health Behavior Inventory–Number of Points		Classification	n	%
Female	Men			
24–77	24–71	Low	146	24.87
78–91	72–86	Average	245	41.74
92–120	87–120	High	196	33.39

**Table 3:** Characteristics of Health Behavior Inventory (n = 587).

n	Moderate	SD	Median	Min	Max	Q1	Q3
587	84.88	11.63	85	45	119	76	93

n—numbers; SD—standard deviation; Min—minimum; Max—maximum; Q1, Q3—quartiles.

**Table 4:** The level of health behaviors division into subscales.

HBI	n	Moderate	SD	Median	Min	Max	Q1	Q3
PEH	587	3.53	0.63	3.67	1.5	5	3.17	4
PB	587	3.62	0.62	3.67	1.83	5	3.17	4
PMA	587	3.61	0.58	3.67	1.67	5	3.17	4
HP	587	3.39	0.62	3.33	1.17	5	3	3.83

n—numbers; SD—standard deviation; Min—minimum; Max—maximum; Q1, Q3—quartiles.

**Table 5:** Predicators of health behavior (HBI). Univariate and multivariate analysis.

Variable		Univariate Model				Multivariate Model			
		Parameter	95%CI		*p*	Parameter	95%CI		*p*
Age	[years]	0.021	−0.081	0.123	0.693	0.292	−0.085	0.669	0.13
Sex	Female	ref.				ref.			
	Male	−0.665	−3.053	1.722	0.585	−1.264	−3.917	1.389	0.351
Work experience	[years]	−0.003	−0.102	0.096	0.951	−0.28	−0.651	0.091	0.139
Education	Basic nursing education	ref.				ref.			
	Bachelor	1.78	−1.639	5.199	0.308	2.18	−1.588	5.949	0.257
	Master’s degree	−0,022	−2.726	2.682	0.987	0.447	−2.605	3.499	0.774
Place of work: PHC	No	ref.				ref.			
	Yes	0.49	−1.413	2.394	0.614	1.216	−2.113	4.545	0.474
Place of work: hospital	No	ref.				ref.			
	Yes	−0.808	−2.771	1.155	0.42	0.967	−2.864	4.799	0.621
Place of work: IHI	No	ref.				ref.			
	Yes	1.455	−2.157	5.066	0.43	2.776	−1.524	7.076	0.206
Place of work: OSC	No	ref.				ref.			
	Yes	0.193	−3.065	3.451	0.908	2.062	−2.395	6.52	0.365
Place of work: hospice	No	ref.				ref.			
	Yes	0.857	−2.485	4.199	0.616	1.986	−2.385	6.358	0.374
Place of work: HCC/SWH	No	ref.				ref.			
	Yes	0.725	−2.019	3.468	0.605	1.918	−2,028	5,864	0,341
Place of work: Long-term care	No	ref.				ref.			
	Yes	0.363	−2.103	2.828	0.773	1.776	−2.025	5.577	0.36
Place of work: private sector	No	ref.				ref.			
	Yes	0.026	−2.519	2.572	0.984	1.042	−2.604	4.688	0.576
Place of work: Nursing home/resort	No	ref.				ref.			
	Yes	−1.353	−5.132	2.426	0.483	−0.842	−5.511	3.826	0.724
More than one full-time job		−0.169	−2.158	1.819	0.867	−1.823	−5.757	2.11	0.364
Shift work and night duty	No	ref.				ref.			
	Yes	−0.547	−3.4	2.305	0.707	−0.983	−4.247	2.282	0.555
BMI	[kg/m^2^]	0.016	−0.086	0.118	0.763				
Self-assessment of health	Very good	ref.				ref.			
	Good	−0.412	−3.173	2.349	0.77	−0.574	−3.38	2.232	0.689
	No opinion	−0.678	−4.059	2.703	0.694	−0.99	−4.443	2.463	0.574
	Bad	0.584	−4.109	5.277	0.808	0.189	−4.593	4.971	0.938
Participation in preventive examinations other than obligatory	No	ref.				ref.			
	Yes	−0.513	−2.717	1.69	0.648	−0.198	−2.526	2.13	0.868
Chronic diseases	No	ref.				ref.			
	Yes	−0.037	−2.344	2.269	0.975	−0.075	−2.505	2.356	0.952

PHC—Primary Health Care; IHI—Independent Healthcare Institution; OSC—Outpatients Specialist Care; HCC—Health Care Center; SWH—Social Welfare Home; Long-term care.

**Table 6:** Sedentary time in the study group.

n	Average	SD	Median	Min	Max	Q1	Q3
587	5.62	1.77	5.43	1.71	11.29	4.29	6.86

n—numbers; SD—standard deviation; Min—minimum; Max—maximum; Q1, Q3—quartiles.

**Table 7:** Predictors of nurses’ sedentary behaviors (n = 587).

Variables		Univariate Model				Multivariate Model			
		Parameter	95%CI		*p*	Parameter	95%CI		*p*
Age	[years]	0.01	−0.006	0.025	0.211	−0.007	−0.063	0.05	0.821
Sex	Female	ref.				ref.			
	Male	−0.573	−0.933	−0.213	0.002 *	−0.489	−0.886	−0.093	0.016 *
Work experience	[years]	0.011	−0.004	0.026	0.148	0.01	−0.045	0.065	0.27
Education	Basic nursing education	ref.				ref.			
	Bachelor	−0.05	−0.57	0.47	0.849	0.058	−0.505	0.621	0.841
	Master’s degree	−0.21	−0.621	0.201	0.317	−0.086	−0.542	0.37	0.712
Place of work: PHC	No	ref.				ref.			
	Yes	−0.151	−0.44	0.138	0.307	−0.194	−0.691	0.303	0.445
Place of work: Hospital	No	ref.				ref.			
	Yes	−0.106	−0.405	0.192	0.485	−0.148	−0.721	0.424	0.612
Place of work: IHI	No	ref.				ref.			
	Yes	−0.305	−0.854	0.244	0.276	−0.199	−0.842	0.443	0.543
Place of work: OSC	No	ref.				ref.			
	Yes	−0.014	−0.509	0.481	0.957	0.094	−0.572	0.76	0.782
Place of work: Hospice	No	ref.				ref.			
	Yes	−0.195	−0.703	0.312	0.451	−0.014	−0.667	0.639	0.967
Place of work: HCC/SWH	No	ref.				ref.			
	Yes	0.376	−0.04	0.792	0.077	0.423	−0.166	1.013	0.16
Place of work: Long-term care	No	ref.				ref.			
	Yes	−0.155	−0.53	0.219	0.417	0.16	−0.408	0.728	0,.81
Place of work: Private sector	No	ref.				ref.			
	Yes	−0.065	−0.452	0.322	0.743	0.082	−0.463	0.626	0.768
Place of work: Nursing home/resort	No	ref.				ref.			
	Yes	0.111	−0.464	0.685	0.706	0.185	−0.512	0.882	0.604
More than one full-time job		−0.38	−0.68	−0.079	0.014 *	−0.513	−1.1	0.075	0.088
Shift work and night duty	No	ref.				ref.			
	Yes	0.103	−0.331	0.536	0.643	0.251	−0.236	0.739	0.313
BMI	[kg/m^2^]	−0.007	−0.022	0.009	0.401				
Self-assessment of health	Very good	ref.				ref.			
	Good	−0.108	−0.526	0.311	0.614	−0.062	−0.481	0.357	0.771
	No opinion	0.172	−0.34	0.685	0.51	0.228	−0.288	0.743	0.387
	Bad	0.419	−0.292	1.13	0.249	0.396	−0.319	1.11	0.278
Participation in preventive examinations other than obligatory	No	ref.				ref.			
	Yes	−0.042	−0.377	0.292	0.804	−0.028	−0.376	0.319	0.873
Chronic diseases	No	ref.				ref.			
	Yes	−0.087	−0.437	0.264	0.628	−0.092	−0.455	0.271	0.618

* Statistically significant relationship (*p* < 0.05); PHC—Primary Health Care; IHI—Independent Healthcare Institution; OSC—Outpatients Specialist Care; HCC—Health Care Center; SWH—Social Welfare Home; Long-term care.

**Table 8:** Correlation between sedentary time and the level of health behaviors.

Health Behavior Inventory (HBI)	Sedentary Time
	Spearman’s Correlation Coefficient
HBI overall score	r = −0.056; *p* = 0.172
PEH	r = −0.025; *p* = 0.55
PB	r = 0; *p* = 0.996
PMA	r = −0.132; *p* = 0.001 *
HP	r = −0.016; *p* = 0.706

* statistically significant relationship (*p* < 0.05).

## Data Availability

The data presented in this study are available on reasonable request from the corresponding author: abartosiewicz@ur.edu.pl.

## Supplementary Materials

The following supporting information can be downloaded at: https://www.mdpi.com/article/10.3390/nu15061312/s1, Table S1: Predictors of Proper Eating Habits subscale. Univariate and multivariate analysis. Table S2: Predictors of Preventive Behavior subscale. Univariate and multivariate analysis. Table S3: Predictors of Positive Mental Attitude subscale. Univariate and multivariate analysis. Table S4: Predictors of Health Practices subscale. Univariate and multivariate analysis.
