# Supplementary material for: Health and Sedentary Behaviors within Polish Nurses: A Cross-Sectional Study
Source: Nutrients. 2023 Mar 7;15(6):1312. doi: 10.3390/nu15061312 (PMC10058938; doi:10.3390/nu15061312)
Supplement: Supplementary file 1 [file nutrients-15-01312-s001.zip › nutrients-2237653-supplementary.pdf]

Table—Supplementary Table S1. Preditators of Proper Eating Habits subscale. Univariate and multivariate analysis

Variable		Univariate model				Multivariate model			
		**Parameter**	**95%CI**		***p***	**Parameter**	**95%CI**		***p***
Age	[years]	0.002	-0.004	0.007	0.483	-0.001	-0.021	0.019	0.921
Sex	Female	ref.				ref.			
	Male	-0.083	-0.211	0.045	0.205	-0.075	-0.216	0.066	0.296
Work experience	[years]	0.002	-0.003	0.007	0.429	0.005	-0.015	0.024	0.646
Education	Basic nursing education	ref.				ref.			
	Bachelor	0.249	0.066	0.432	0.008 *	0.314	0.114	0.514	0.002 *
	Master degree	0.091	-0.054	0.235	0.219	0.158	-0.004	0.32	0.057
Place of work: PHC	No	ref.				ref.			
	Yes	0.014	-0.088	0.116	0.789	0.114	-0.062	0.291	0.205
Place of work: hospital	No	ref.				ref.			
	Yes	-0.053	-0.159	0.052	0.323	0.097	-0.106	0.3	0.35
Place of work: IHI	No	ref.				ref.			
	Yes	0.132	-0.062	0.326	0.182	0.239	0.011	0.467	0.041 *
Place of work: OSC	No	ref.				ref.			
	Yes	-0.052	-0.227	0.123	0.561	0.076	-0.16	0.313	0.528
Place of work: hospice	No	ref.				ref.			
	Yes	0.058	-0.122	0.238	0.527	0.18	-0.052	0.412	0.128
Place of work: HCC/SWH	No	ref.				ref.			
	Yes	0.075	-0.072	0.223	0.317	0.191	-0.019	0.4	0.075

Variable		Univariate model				Multivariate model			
		**Parameter**	**95%CI**		***p***	**Parameter**	**95%CI**		***p***
Place of work: Long-term care	No	ref.				ref.			
	Yes	-0.002	-0.134	0.131	0.979	0.153	-0.049	0.355	0.137
Place of work: private sector	No	ref.				ref.			
	Yes	0.026	-0.111	0.163	0.711	0.161	-0.033	0.354	0.104
Place of work: Nursing home/resort	No	ref.				ref.			
	Yes	0.111	-0.092	0.314	0.284	0.213	-0.035	0.461	0.092
More than one full-time job			-0.12	0.094	0.807	-0.206	-0.415	0.003	0.054
Shift work and night duty	No	ref.				ref.			
	Yes	0.012	-0.141	0.165	0.879	-0.031	-0.204	0.143	0.728
BMI	[kg/m²]	0.001	-0.005	0.006	0.857				
Self-assesment of health	Very good	ref.				ref.			
	Good	-0.094	-0.243	0.054	0.213	-0.101	-0.25	0.048	0.186
	No opinion	-0.094	-0.276	0.087	0.31	-0.12	-0.303	0.064	0.202
	Bad	-0.089	-0.341	0.163	0.49	-0.118	-0.372	0.135	0.361
Participation in preventive examinations other than obligatory	No	ref.				ref.			
	Yes	0.042	-0.077	0.16	0.491	0.05	-0.073	0.174	0.424
Chronic deseases	No	ref.				ref.			
	Yes	0.02	-0.104	0.144	0.753	0.017	-0.112	0.146	0.791

* Statistically significant relationship ($p < 0.05$); PHC – Primary Health Care; IHI - Independent Healthcare Institution; OSC – Outpatients Specialist Care; HCC – Health Care Center; SWH - Social Welfare Home; Long-term care.

Table—Supplementary Table S2. Preditators of Preventive Behavior subscale. Univariate and multivariate analysis

Variable		Univariate model				Multivariate model			
		Parameter	95%CI		*p*	Parameter	95%CI		*p*
Age	[years]	0.002	-0.004	0.007	0.49	0.008	-0.013	0.028	0.46
Sex	Female	ref.				ref.			
	Male	-0.06	-0.188	0.068	0.357	-0.075	-0.217	0.067	0.299
Work experience	[years]	0.001	-0.004	0.007	0.641	-0.007	-0.027	0.013	0.479
Education	Basic nursing education	ref.				ref.			
	Bachelor	-0.015	-0.198	0.168	0.87	0.003	-0.199	0.204	0.978
	Master degree	-0.079	-0.224	0.066	0.284	-0.066	-0.229	0.097	0.426
Place of work: PHC	No	ref.				ref.			
	Yes	0.029	-0.073	0.131	0.578	0.025	-0.153	0.203	0.78
Place of work: hospital	No	ref.				ref.			
	Yes	-0.075	-0.18	0.03	0.16	-0.022	-0.227	0.182	0.83
Place of work: IHI	No	ref.				ref.			
	Yes	0.087	-0.106	0.28	0.377	0.14	-0.089	0.37	0.232
Place of work: OSC	No	ref.				ref.			
	Yes	0.048	-0.126	0.222	0.59	0.109	-0.129	0.347	0.371
Place of work: hospice	No	ref.				ref.			
	Yes	0.004	-0.175	0.183	0.967	0.034	-0.199	0.268	0.774
Place of work: HCC/SWH	No	ref.				ref.			

Variable		Univariate model				Multivariate model			
		**Parameter**	**95%CI**		***p***	**Parameter**	**95%CI**		***p***
	Yes	0.005	-0.142	0.152	0.947	0.033	-0.178	0.244	0.762
Place of work: Long-term care	No	ref.				ref.			
	Yes	0.073	-0.058	0.205	0.276	0.126	-0.077	0.329	0.224
Place of work: private sector	No	ref.				ref.			
	Yes	0.022	-0.114	0.158	0.755	0.057	-0.138	0.251	0.57
Place of work: Nursing home/resort	No	ref.				ref.			
	Yes	-0.064	-0.267	0.138	0.533	-0.063	-0.313	0.186	0.62
More than one full-time job			-0.106	0.107	0.989	-0.07	-0.28	0.141	0.516
Shift work and night duty	No	ref.				ref.			
	Yes	-0.029	-0.182	0.124	0.71	-0.02	-0.194	0.155	0.826
BMI	[kg/m²]	0.001	-0.004	0.007	0.609				
Self-assesment of health	Very good	ref.				ref.			
	Good	-0.019	-0.167	0.129	0.8	-0.027	-0.177	0.123	0.727
	No opinion	-0.069	-0.249	0.112	0.458	-0.09	-0.275	0.095	0.34
	Bad	-0.014	-0.265	0.237	0.913	-0.037	-0.293	0.219	0.777
Participation in preventive examinations other than obligatory	No	ref.				ref.			
	Yes	-0.075	-0.192	0.043	0.215	-0.058	-0.182	0.067	0.364
Chronic deseases	No	ref.				ref.			
	Yes	-0.01	-0.133	0.114	0.875	-0.017	-0.147	0.113	0.797

* Statistically significant relationship ($p < 0.05$); PHC – Primary Health Care; IHI - Independent Healthcare Institution; OSC – Outpatients Specialist Care; HCC – Health Care Center; SWH - Social Welfare Home; Long-term care.

Table—Supplementary Table S3. Preditators of Positive Menthal Attitiude subscale. Univariate and multivariate analysis

Variable		Univariate model				Multivariate model			
		**Parameter**	**95%CI**		***p***	**Parameter**	**95%CI**		***p***
Age	[years]	0.001	-0.004	0.006	0.715	0.026	0.007	0.045	0.007 *
Sex	Female	ref.				ref.			
	Male	0.006	-0.113	0.125	0.923	-0.043	-0.175	0.089	0.522
Work experience	[years]	-0.001	-0.006	0.004	0.662	-0.025	-0.043	-0.007	0.008 *
Education	Basic nursing education	ref.				ref.			
	Bachelor	0.07	-0.1	0.241	0.42	0.088	-0.099	0.275	0.355
	Master degree	0.01	-0.125	0.145	0.881	0.029	-0.122	0.181	0.703
Place of work: PHC	No	ref.				ref.			
	Yes	0.024	-0.071	0.119	0.615	0.023	-0.142	0.188	0.787
Place of work: hospital	No	ref.				ref.			
	Yes	-0.036	-0.134	0.062	0.47	0.01	-0.18	0.2	0.918
Place of work: IHI	No	ref.				ref.			
	Yes	0.001	-0.179	0.181	0.989	0.046	-0.167	0.259	0.673
Place of work: OSC	No	ref.				ref.			
	Yes	0.042	-0.121	0.204	0.616	0.108	-0.114	0.329	0.341
Place of work: hospice	No	ref.				ref.			
	Yes	0.079	-0.087	0.246	0.351	0.09	-0.127	0.307	0.416
Place of work: HCC/SWH	No	ref.				ref.			
	Yes	0.028	-0.109	0.165	0.692	0.058	-0.137	0.254	0.559
Place of work: Long-term care	No	ref.				ref.			

Variable		Univariate model				Multivariate model			
		Parameter	95%CI		*p*	Parameter	95%CI		*p*
	Yes	0.01	-0.113	0.133	0.876	0.027	-0.162	0.215	0.781
Place of work: private sector	No	ref.				ref.			
	Yes	-0.033	-0.16	0.094	0.612	-0.016	-0.197	0.165	0.862
Place of work: Nursing home/resort	No	ref.				ref.			
	Yes	-0.142	-0.331	0.046	0.138	-0.158	-0.39	0.074	0.182
More than one full-time job			-0.115	0.084	0.758	-0.039	-0.234	0.156	0.695
Shift work and night duty	No	ref.				ref.			
	Yes	-0.039	-0.181	0.103	0.59	-0.046	-0.208	0.116	0.578
BMI	[kg/m²]	0	-0.005	0.005	0.956				
Self-assesment of health	Very good	ref.				ref.			
	Good	0.037	-0.101	0.174	0.601	0.029	-0.11	0.168	0.683
	No opinion	-0.002	-0.171	0.167	0.982	-0.006	-0.178	0.165	0.944
	Bad	-0.015	-0.249	0.219	0.903	-0.029	-0.266	0.208	0.811
Participation in preventive examinations other than obligatory	No	ref.				ref.			
	Yes	-0.015	-0.125	0.095	0.786	-0.004	-0.119	0.112	0.948
Chronic deseases	No	ref.				ref.			
	Yes	-0.015	-0.13	0.1	0.797	-0.022	-0.142	0.099	0.723

* Statistically significant relationship ($p < 0.05$); PHC – Primary Health Care; IHI - Independent Healthcare Institution; OSC – Outpatients Specialist Care; HCC – Health Care Center; SWH - Social Welfare Home; Long-term care.

Table—Supplementary Table S4. Preditators of Health Practices subscale. Univariate and multivariate analysis

Variable		Univariate model				Multivariate model			
		Parameter	95%CI		*p*	Parameter	95%CI		*p*
Age	[years]	-0.001	-0.007	0.004	0.596	0.016	-0.004	0.036	0.125
Sex	Female	ref.				ref.			
	Male	0.027	-0.101	0.155	0.677	-0.016	-0.158	0.126	0.824
Work experience	[years]	-0.003	-0.008	0.002	0.29	-0.019	-0.039	0.001	0.064
Education	Basic nursing education	ref.				ref.			
	Bachelor	-0.009	-0.193	0.174	0.923	-0.044	-0.246	0.157	0.666
	Master degree	-0.028	-0.174	0.117	0.702	-0.051	-0.214	0.112	0.542
Place of work: PHC	No	ref.				ref.			
	Yes	0.014	-0.088	0.117	0.781	0.04	-0.138	0.218	0.659
Place of work: hospital	No	ref.				ref.			
	Yes	0.03	-0.075	0.135	0.577	0.077	-0.128	0.282	0.464
Place of work: IHI	No	ref.				ref.			
	Yes	0.022	-0.172	0.216	0.824	0.037	-0.193	0.267	0.752
Place of work: OSC	No	ref.				ref.			
	Yes	-0.005	-0.18	0.17	0.954	0.051	-0.187	0.29	0.674
Place of work: hospice	No	ref.				ref.			
	Yes	0.002	-0.177	0.181	0.984	0.027	-0.207	0.261	0.823
Place of work: HCC/SWH	No	ref.				ref.			
	Yes	0.014	-0.133	0.161	0.852	0.039	-0.172	0.25	0.719

Variable		Univariate model				Multivariate model			
		Parameter	95%CI		*p*	Parameter	95%CI		*p*
Place of work: Long-term care	No	ref.				ref.			
	Yes	-0.021	-0.153	0.111	0.759	-0.01	-0.213	0.194	0.925
Place of work: private sector	No	ref.				ref.			
	Yes	-0.01	-0.147	0.126	0.884	-0.028	-0.223	0.167	0.778
Place of work: Nursing home/resort	No	ref.				ref.			
	Yes	-0.13	-0.332	0.073	0.209	-0.132	-0.382	0.117	0.3
More than one full-time job			-0.106	0.107	0.991	0.011	-0.2	0.221	0.919
Shift work and night duty	No	ref.				ref.			
	Yes	-0.033	-0.186	0.12	0.672	-0.066	-0.24	0.109	0.462
BMI	[kg/m²]	0.001	-0.005	0.006	0.842				
Self-assesment of health	Very good	ref.				ref.			
	Good	0.006	-0.141	0.154	0.932	0.001	-0.149	0.151	0.989
	No opinion	0.052	-0.129	0.232	0.577	0.05	-0.134	0.235	0.594
	Bad	0.215	-0.036	0.466	0.093	0.217	-0.039	0.473	0.097
Participation in preventive examinations other than obligatory	No	ref.				ref.			
	Yes	-0.038	-0.156	0.081	0.534	-0.022	-0.147	0.103	0.729
Chronic deseases	No	ref.				ref.			
	Yes	-0.001	-0.124	0.123	0.99	0.009	-0.121	0.139	0.887

* Statistically significant relationship ($p < 0.05$); PHC – Primary Health Care; IHI - Independent Healthcare Institution; OSC – Outpatients Specialist Care; HCC – Health Care Center; SWH - Social Welfare Home; Long-term ca
